# D‐Psicose Attenuates Hepatic Steatosis and Inflammatory Injury in HFD/STZ‐Induced T2DM Mice and Is Associated With Alterations in Gut Microbiota, Tryptophan Metabolism, and Hepatic AhR‐Related Signaling

**DOI:** 10.1002/fsn3.72455

**Published:** 2026-10-08

**Authors:** Liang Wu, Xueyun Dong, Wen Sun, Hao Xu, Yunhan Xie, Xuehui Liu, Xinyu Liu, Asmaa Ali, Keke Shao

**Affiliations:** ^1^ Department of Laboratory Medicine, School of Medicine Jiangsu University Zhenjiang China; ^2^ Molecular Medical Research Center Yancheng Clinical Medical College of Jiangsu University (The First People's Hospital of Yancheng) Yancheng China; ^3^ Jiangsu Key Laboratory of Medical Science and Laboratory Medicine, School of Medicine Jiangsu University Zhenjiang Jiangsu China; ^4^ Critical Care Medicine Jurong Hospital Affiliated to Jiangsu University Zhenjiang China; ^5^ Department of Laboratory Medicine The Yancheng Clinical College of Xuzhou Medical University (The First People's Hospital of Yancheng) Yancheng China

**Keywords:** aryl hydrocarbon receptor, D‐psicose, gut microbiota, liver injury, tryptophan metabolism, Type 2 diabetes

## Abstract

D‐psicose (DPS) is a rare sugar with reported metabolic benefits, but its effects on diabetes‐associated liver abnormalities and related gut–liver changes remain insufficiently defined. Here, DPS was evaluated in mice with Type 2 diabetes mellitus induced by a high‐fat diet and streptozotocin. Low‐ and high‐dose DPS interventions were assessed through serum biochemistry, liver histology, inflammatory cytokine measurements, intestinal barrier evaluation, hepatic molecular analysis, 16S rRNA gene sequencing, targeted quantification of tryptophan metabolites, and untargeted serum metabolomics. Relative to untreated diabetic mice, DPS‐treated animals showed improved serum lipid profiles, lower ALT and AST activities, reduced circulating inflammatory cytokines, and less hepatic lipid deposition. DPS treatment was also accompanied by improved intestinal morphology, increased ZO‐1 and Occludin expression, and lower circulating lipopolysaccharide concentrations. In the high‐dose group, gut microbial community composition differed from that of untreated diabetic mice, with relative increases in Akkermansia and Lactobacillus. In parallel, DPS treatment was associated with increased serum tryptophan and indole‐3‐propionic acid concentrations, lower kynurenine concentrations and Kyn/Trp ratios, reduced hepatic TDO2 expression, increased AhR expression, and lower NF‐κB p65 phosphorylation. Overall, DPS improved metabolic and liver‐related abnormalities in diabetic mice and was accompanied by coordinated changes in intestinal barrier‐related indices, gut microbial composition, tryptophan metabolites, and hepatic AhR/NF‐κB‐related markers. As microbiota‐directed and AhR‐targeted intervention experiments were not conducted, these observations remain associative and do not demonstrate a causal gut microbiota–tryptophan–AhR mechanism. Moreover, as these observations were obtained in a single HFD/STZ‐induced mouse model, their relevance to the heterogeneity and natural course of human T2DM‐associated MASLD requires confirmation in additional preclinical models and clinical studies.

## Graphical abstract

DPS functions as a regulator of the Gut‐Liver Axis to treat T2DM/MASLD. This study elucidates the molecular mechanism by which DPS mitigates diabetic liver injury. T2DM patients often exhibit disordered tryptophan metabolism and suppressed Aryl hydrocarbon Receptor (AhR) signaling. DPS intervention successfully reverses this by inhibiting hepatic TDO2 overexpression and facilitating the conversion of tryptophan into IPA. The restored IPA acts as a ligand to activate the hepatic AhR pathway, which subsequently inhibits NF‐κB‐mediated inflammation. Confirmed by metabolomics and molecular biology, DPS targets the “Gut Microbiota‐Tryptophan Metabolism‐AhR” axis, offering a promising nutritional adjunct therapy for diabetes‐associated liver damage.

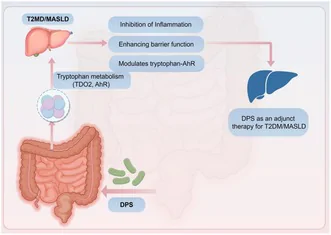

## 1 Introduction

Diabetes mellitus constitutes a major worldwide health crisis. Among its various co‐morbidities, metabolic dysfunction‐associated steatotic liver disease (MASLD) is particularly widespread but often fails to garner sufficient clinical attention. Epidemiological data indicate that MASLD affects the majority of individuals with diabetes, with incidence rates outstripping those of diabetic kidney disease (Younossi et al. 2019). The frequently asymptomatic nature of early‐stage MASLD leads to missed diagnoses, making it one of the most overlooked complications and necessitating more aggressive strategies for early detection and management (Portillo‐Sanchez et al. 2015).

Characterized by hepatic fat overload, inflammation, and fibrosis, the development of MASLD is driven by obesity and insulin resistance. The clinical trajectory of the disease is concerning; without intervention, it may progress to steatohepatitis and cirrhosis, drastically increasing the risk of liver cancer (Dulai et al. 2017). Beyond liver pathology, MASLD aggravates diabetes severity and correlates independently with cardiovascular events and mortality, posing a severe health burden (Targher et al. 2016). To investigate diabetes‐associated metabolic and hepatic abnormalities in vivo, a high‐fat diet combined with low‐dose streptozotocin (HFD/STZ) is commonly used in rodents. This model combines diet‐related insulin resistance with STZ‐induced β‐cell injury and reproduces selected features of T2DM‐associated metabolic dysfunction, including hyperglycemia, dyslipidemia, oxidative stress, and hepatic lipid accumulation (Hazem et al. 2022; Racine et al. 2024). However, it does not fully capture the heterogeneity, disease duration, environmental influences, or complete histological spectrum of human T2DM‐associated MASLD. Accordingly, the HFD/STZ model provides a useful preclinical system for examining treatment‐associated biological effects, whereas confirmation in complementary models and clinical studies is needed before translational conclusions can be drawn. In the present study, this phenotype is referred to as HFD/STZ‐induced T2DM with hepatic steatosis and inflammatory injury. This terminology describes the metabolic and liver‐related features directly evaluated in the model and does not imply that the model is equivalent to human MASLD.

D‐Psicose (DPS/D‐allulose) is a low‐calorie epimer of fructose with approximately 70% of the sweetness of sucrose and limited caloric contribution. Previous experimental and clinical studies have suggested that DPS may influence postprandial glycemic responses and metabolic indices (Noronha et al. 2018). Experimental evidence has also associated DPS exposure with changes in hepatic lipid metabolism, insulin resistance, oxidative stress, and inflammatory status (Bae et al. 2023; Han et al. 2018; Hossain et al. 2015). Nevertheless, these findings do not establish DPS as a treatment for T2DM‐associated MASLD, and its effects on diabetes‐associated liver abnormalities require further investigation. The present study therefore examined the effects of DPS in an HFD/STZ‐induced T2DM mouse model. The aim was to characterize treatment‐associated changes in liver‐related phenotypes and in selected intestinal, microbial, metabolic, and hepatic signaling indices, rather than to make direct clinical or therapeutic inferences.

Gut microbiota‐derived tryptophan metabolites have been reported to interact with host immune and metabolic signaling pathways, including AhR‐related signaling. However, whether changes in microbial composition, circulating tryptophan metabolites, and hepatic AhR‐related markers are causally linked during DPS treatment remains unclear.

In the present study, we evaluated the effects of DPS in HFD/STZ‐induced T2DM mice and examined treatment‐associated changes in gut microbial composition, intestinal barrier‐related indices, serum tryptophan metabolites, hepatic TDO2 and AhR expression, and NF‐κB signaling. This study was designed to identify coordinated biological changes and generate mechanistic hypotheses; it was not designed to establish causal relationships among gut microbiota, tryptophan metabolism, AhR signaling, and hepatic protection.

## 2 Materials and Methods

### 2.1 Animal Maintenance, T2DM Model Induction, and DPS Intervention

Eight‐week‐old male C57BL/6J mice were obtained from Jiangsu Wukong Biotechnology Co. Ltd. (Nanjing, China). Mice were acclimatized for 1 week under specific pathogen‐free conditions at 23°C ± 2°C, 60% ± 5% relative humidity, and a 12‐h light/dark cycle, with free access to food and water. All animal procedures were reviewed and approved by the Animal Ethics Committee of Jiangsu University (Approval No. UJS‐IACUCAP‐2024032261).

Following acclimatization, mice were randomly allocated to the normal control (NC) group (*n* = 6) or the diabetes‐induction cohort (*n* = 18). Mice in the diabetes‐induction cohort were fed a high‐fat diet containing 60% fat (Xietong Co. Ltd., China; #HFM001) for 4 weeks. Hyperglycemia was then induced by intraperitoneal injection of streptozotocin (STZ; 40 mg/kg/day; Sigma‐Aldrich, USA) in 0.1 M citrate buffer (pH 4.5) for 5 consecutive days. NC mice received the citrate‐buffer vehicle. One week after the final STZ injection, fasting blood glucose was measured, and mice with a value > 11.1 mmol/L were considered diabetic.

Diabetic mice were randomly assigned to the untreated T2DM group, low‐dose DPS group (DPSL), or high‐dose DPS group (DPSH), with six mice in each group. DPS was administered in drinking water at concentrations of 2.5% (w/v) in the DPSL group and 5.0% (w/v) in the DPSH group for 12 weeks. Mice in the NC and T2DM groups received drinking water without DPS. The NC group received a standard diet, whereas the T2DM, DPSL, and DPSH groups continued to receive the high‐fat diet throughout the intervention period. Body weight was recorded weekly.

The overall experimental timeline consisted of 1 week of acclimatization, 4 weeks of high‐fat diet feeding, 5 consecutive days of STZ administration, 1 week for diabetes confirmation, and 12 weeks of DPS or vehicle intervention. At the end of the experiment, mice were anesthetized with sevoflurane and euthanized by cervical dislocation after the absence of pain reflexes had been confirmed. Blood, liver tissue, intestinal tissue, and colonic contents were collected for subsequent analyses.

### 2.2 Determination of Serum Markers and Metabolites

Following blood collection, serum was separated by centrifugation (3000 rpm, 15 min) after a 2‐h clotting period. Lipid (TC, TG, LDL‐C, HDL‐C) and hepatic injury biomarkers (ALT, AST) were assayed utilizing a Hitachi 7180 chemistry analyzer (Ardekani et al. 2023). Systemic endotoxemia was assessed by measuring LPS levels with a commercial LAL kit (#H245, Xiamen Bioendo) (Zhang et al. 2025). The UPLC‐MS/MS analysis for tryptophan metabolites (Trp, Kyn, IPA) was carried out by the Department of Clinical Laboratory, Affiliated Women's Hospital of Jiangnan University. Briefly, serum proteins were precipitated using 4‐fold volumes of cold methanol (0.1% formic acid). After centrifugation at 12,000 × g (4°C), the supernatant was analyzed on an ACQUITY UPLC HSS T3 column (Waters Corp.). The mobile phase comprised 0.1% formic acid in water (A) and acetonitrile (B) at 0.3 mL/min under a gradient elution profile (Hossen et al. 2025). Mass spectrometric detection utilized positive ESI and MRM modes for optimized selectivity. Absolute quantification was performed using external standard calibration curves.

### 2.3 Pathological Assessment

Histological processing and staining were carried out by the Department of Pathology, The First People's Hospital of Yancheng. Liver tissues were divided for two preparation methods: PFA‐fixed paraffin embedding (4 μm sections) for structural analysis and OCT‐embedded cryosectioning (8 μm sections) for lipid detection (Ge et al. 2010). Paraffin sections underwent standard H&E staining. Briefly, rehydrated sections were stained with hematoxylin (Solarbio) for 5 min and eosin for 2 min. After dehydration and mounting, the architecture and inflammatory status of the liver lobules were observed. For Oil Red O staining, frozen sections were air‐dried and stained with Oil Red O solution (Solarbio) for 10 min to visualize neutral triglycerides (Catta‐Preta et al. 2011). Differentiation was performed using 60% isopropanol, followed by nuclear counterstaining with hematoxylin (1 min). Red‐stained lipid droplets were visualized under a light microscope after mounting with an aqueous medium. Histological assessment and image‐based quantification were performed by investigators blinded to group allocation.

### 2.4 Immunohistochemical Analysis of Intestinal Tight Junctions

To evaluate intestinal barrier integrity, 4 μm paraffin‐embedded intestinal sections were subjected to immunohistochemical staining for ZO‐1 and Occludin. Antigen retrieval was achieved via citrate buffer heating after deparaffinization. To minimize background, slides were treated with 3% H_2_O_2_ and normal goat serum. Samples were incubated overnight (4°C) with rabbit monoclonal antibodies targeting ZO‐1 or Occludin (Proteintech). Detection was carried out using an HRP‐conjugated secondary antibody and DAB substrate system. Nuclei were counterstained with hematoxylin. After mounting, the intensity and localization of the brownish‐yellow positive signals for ZO‐1 and Occludin were assessed via light microscopy.

### 2.5 QPCR and Western Blotting

RNA was extracted from liver tissue (TRIzol, Vazyme) and reverse‐transcribed to cDNA (HiScript II Kit, Vazyme). Real‐time PCR was carried out using ChambQ SYBR Mix (Vazyme) and specific primers (Suzhou Genwiz, in Methods S1 and Table S1). Relative expression was computed using the 2^−ΔΔCt^ formula (Ma et al. 2021). Western blot analysis was performed using liver lysates. Detailed methodological information is provided in Methods S2 and Table S2.

### 2.6 Integrated 16S rRNA Sequencing and Metabolomic Profiling

To investigate the gut‐metabolism interplay, we analyzed colonic contents and serum samples (Agus et al. 2021).

#### 2.6.1 Gut Microbiota Analysis

Colonic‐content samples were snap‐frozen in liquid nitrogen and stored at −80°C until DNA extraction. Sequencing was performed by Shenzhen Microbiome Tech Co. Ltd. (Shenzhen, China). The V3–V4 hypervariable regions of the bacterial 16S rRNA gene were amplified using the primers 338F (5′‐ACTCCTACGGGAGGCAGCAG‐3′) and 806R (5′‐GGACTACHVGGGTWTCTAAT‐3′) and sequenced on an Illumina NovaSeq platform using paired‐end reads. Raw reads were processed in QIIME 2 (version 2023.5). After demultiplexing and quality filtering, paired‐end reads were denoised, merged, and screened for chimeric sequences using the DADA2 plugin, generating amplicon sequence variants (ASVs). Taxonomic assignment was performed using a trained Naïve Bayes classifier against the SILVA reference database (release 138.1). Greengenes was not used in the present analysis. Alpha‐diversity indices, including Shannon and Simpson indices, were calculated from the ASV table. Beta‐diversity was evaluated using Bray–Curtis distance matrices and visualized by principal coordinates analysis (PCoA). Group‐level differences in microbial community composition were assessed by PERMANOVA. Differentially represented taxa were explored using LEfSe with an LDA score threshold of > 2.0 and a nominal *p* value < 0.05. Because 16S rRNA gene sequencing provides taxonomic rather than direct functional information, the analysis was used to describe treatment‐associated compositional differences and not to infer the metabolic capacity of individual taxa.

#### 2.6.2 Untargeted Serum Metabolomics

For the metabolomics analysis, the high‐dose DPS group (DPSH) is hereafter referred to as the DPS group. Serum samples were analyzed by UPLC‐Q‐TOF‐MS in both positive‐ and negative‐electrospray ionization modes. Raw data processing, including peak detection, alignment, normalization, and metabolite annotation, was performed using Progenesis QI version 2.3 (Nonlinear Dynamics, Newcastle upon Tyne, UK). Pooled quality‐control samples were included throughout the analytical sequence to monitor system stability. Unsupervised principal component analysis (PCA) was used to visualize global metabolic variation and identify potential outliers. Pairwise orthogonal partial least‐squares discriminant analysis (OPLS‐DA) models were constructed in SIMCA‐P version 17.0 for the NC versus T2DM and DPS versus T2DM comparisons in both positive‐ and negative‐ion modes. Model performance was assessed using seven‐fold cross‐validation and 200‐permutation testing. The cumulative explained variance of the predictor matrix [R^2^X(cum)], cumulative explained variance of the response matrix [R^2^Y(cum)], cumulative predictive ability [Q^2^(cum)], and permutation‐test *p* values are reported in Methods S3, Table S3, and Figure S1. Differential metabolites were selected based on a variable importance in projection score > 1.0, fold change > 1.5 or < 0.67, and an FDR‐adjusted *q* value < 0.05 using the Benjamini–Hochberg procedure.

#### 2.6.3 Pathway Analysis

Significant metabolites were mapped to the KEGG and HMDB databases via MetaboAnalyst 6.0. Pathway impact was evaluated using topology analysis based on betweenness centrality.

### 2.7 Statistics and Plotting

All quantitative results are reported as the mean ± SD from the independent animals per experimental group. Distributional normality and variance equality were examined with the Shapiro–Wilk and Levene tests, respectively. Variables satisfying both criteria were compared across groups by one‐way analysis of variance, with Tukey‐adjusted pairwise comparisons performed when appropriate. Variables that did not satisfy parametric assumptions were evaluated using the Kruskal–Wallis test followed by Dunn's post hoc procedure with Holm correction. For untargeted metabolomics, feature‐wise *p* values were corrected for multiple comparisons with the Benjamini–Hochberg false discovery rate method. Metabolites were designated as differential when they fulfilled all of the following criteria: VIP > 1.0, and *p* < 0.05.

## 3 Results

### 3.1 Impact of DPS on Body Weight and Hepatic Morphology

Successful induction of the T2DM model via HFD/STZ was confirmed by phenotypic changes relative to the NC group. Diabetic mice exhibited a sustained reduction in body weight as the disease progressed. However, DPS intervention effectively curbed this weight loss; notably, the high‐dose group (DPSH) achieved significant weight stabilization compared to untreated diabetic mice (Figure 1A). Macroscopic examination highlighted distinct pathological differences. While livers from the NC group were reddish with a smooth texture, those from the T2DM group appeared enlarged and pale, indicative of severe lipid retention. DPS treatment markedly restored hepatic appearance, with the DPSH group exhibiting a color and texture comparable to healthy controls (Figure 1B). Microscopic analysis using H&E staining further validated these findings. In contrast to the orderly cellular architecture of NC livers, T2DM tissues showed extensive structural disarray, characterized by hepatocellular ballooning and vacuolization. DPS administration significantly reversed these defects, preserving hepatic architecture and reducing inflammatory signs, with superior efficacy observed in the high‐dose cohort (Figure 1C). Furthermore, Oil Red O staining corroborated the severity of steatosis in T2DM mice, revealing massive neutral lipid accumulation. DPS treatment dose‐dependently cleared these lipid deposits, with the DPSH group showing the most profound reduction (Figure 1D). These data collectively demonstrate the potent ability of DPS to correct hepatic lipid dysregulation and alleviate liver pathology in a diabetic context.

**FIGURE 1:**
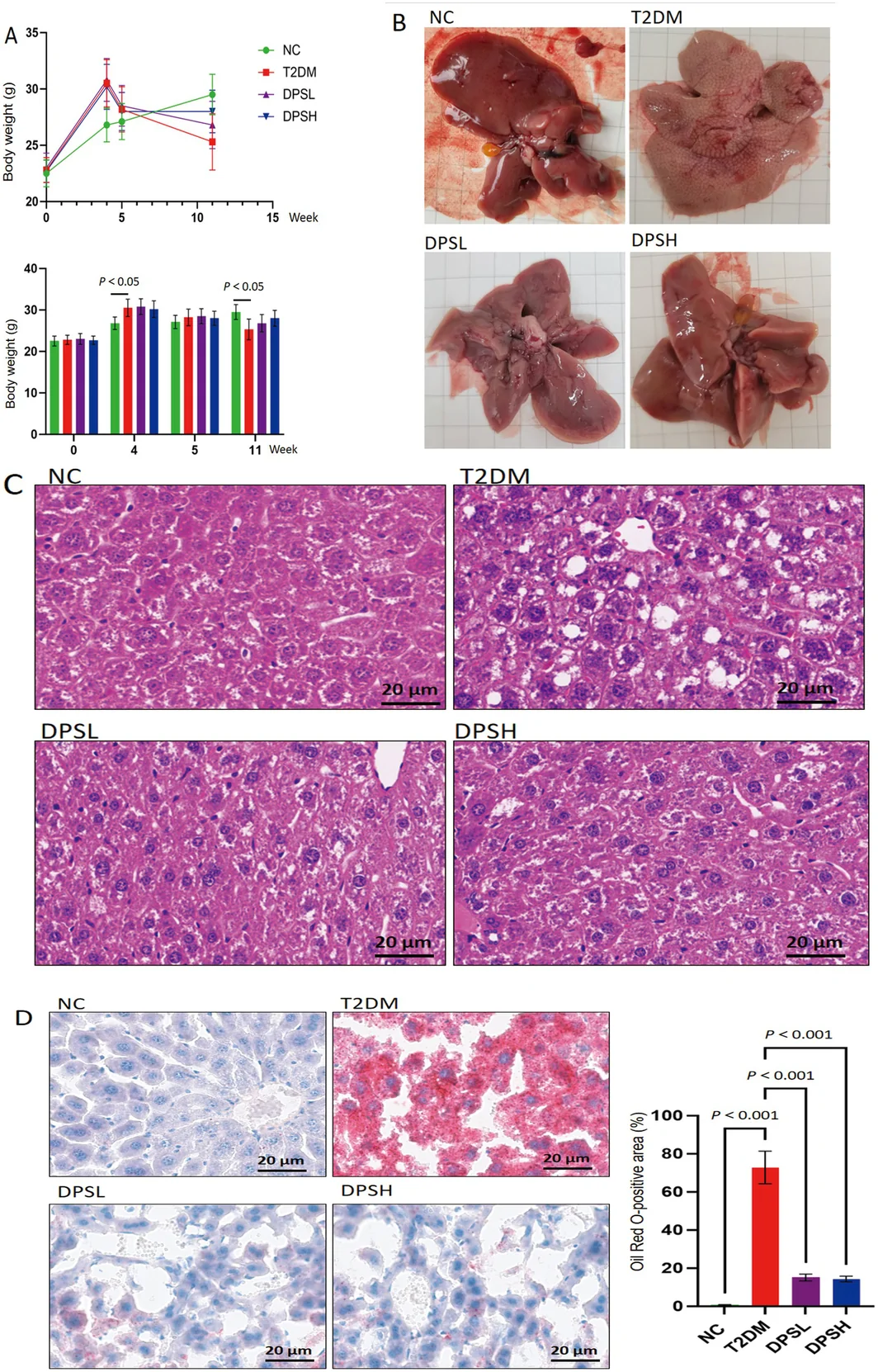
Evaluation of the hepatoprotective effects of DPS (*n* = 6). Experimental diabetes was induced using an HFD and STZ protocol, followed by intervention with low (DPSL) and high (DPSH) doses of DPS. (A) Body weight trends. (B) Gross photographs of explanted livers. (C) Representative H&E‐stained hepatic sections. (D) Representative Oil Red O staining indicating lipid distribution.

### 3.2 Improvement of Lipid Profiles and Inflammatory Status

Compared with the NC group, mice in the T2DM group displayed an unfavorable serum lipid profile, with higher concentrations of TC, TG, and LDL‐C and a lower HDL‐C concentration (Figure 2A–D). Administration of DPS shifted these indices toward the control profile, with the high‐dose regimen producing the larger overall change. T2DM induction was also accompanied by elevations in serum ALT and AST, whereas both indices were lower following DPS treatment, particularly in the DPSH group (Figure 2E,F). In parallel, circulating TNF‐α, IL‐6, and IL‐1β were increased in untreated T2DM mice and decreased after DPS administration (Figure 2G–I). These findings show that DPS exposure coincided with improved lipid‐related, aminotransferase, and inflammatory biochemical profiles in this mouse model.

**FIGURE 2:**
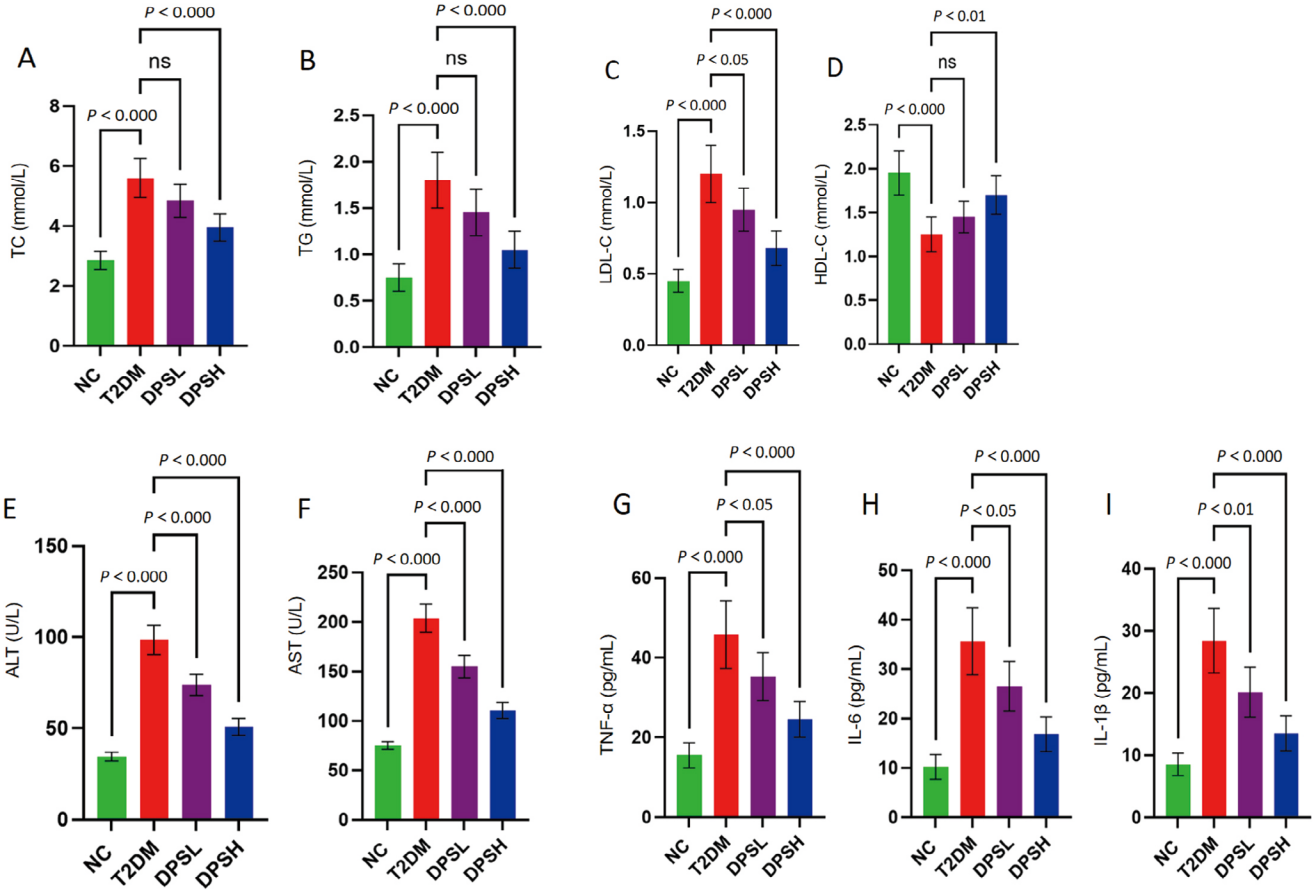
Effects of DPS on serum lipid profiles, hepatic injury markers, and inflammatory cytokines in HFD/STZ‐induced T2DM mice (*n* = 6). Serum levels of (A) TC, (B) TG, (C) LDL‐C, (D) HDL‐C, (E) ALT, (F) AST, (G) TNF‐α, (H) IL‐6, and (I) IL‐1β were measured.

### 3.3 Influence of DPS on the Expression of Key Genes and Proteins in the Liver of T2DM Mice

Figure 3A,B demonstrate that, compared to the NC group, the mRNA expression level of *TDO2*—the rate‐limiting enzyme of the liver Tryptophan‐Kynurenine pathway—was significantly increased, while the expression of *AhR* was markedly decreased in the T2DM group (*p* < 0.05). This suggests a disturbance and aberrant activation of the hepatic tryptophan metabolic pathway under diabetic conditions. Following DPS intervention, this expression trend was reversed in both the DPSL and DPSH groups: *TDO2* expression was downregulated, and *AhR* expression was notably restored, with a more pronounced improvement observed in the high‐dose group (*p* < 0.05). These findings indicate that DPS effectively ameliorates the abnormally activated tryptophan metabolism in the livers of diabetic mice by modulating the TDO2/AhR signaling axis.

**FIGURE 3:**
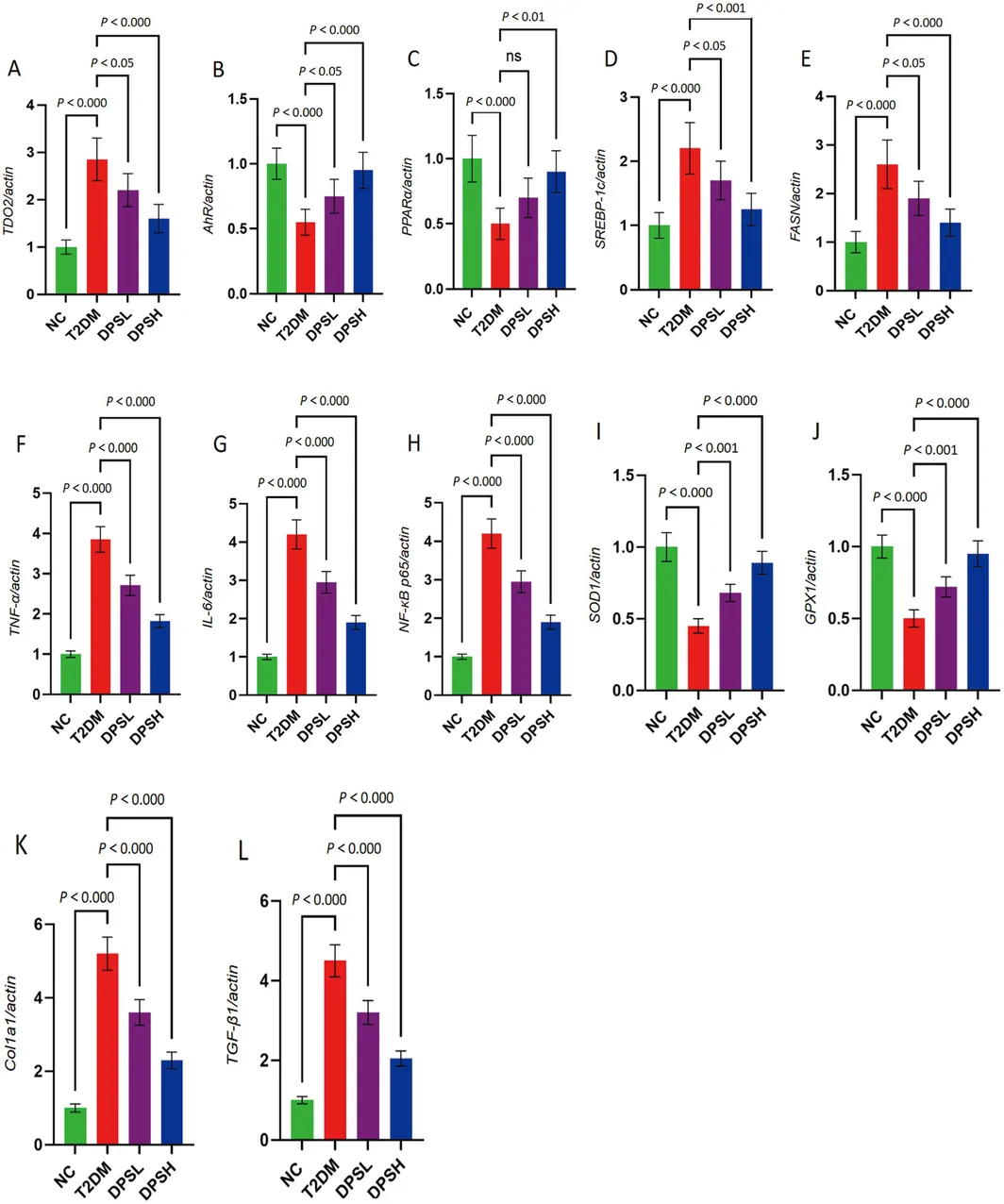
QPCR analysis of key genes in mouse liver tissue (*n* = 6). The relative mRNA expression levels for the following targets are shown: (A) TDO2, (B) AhR, (C) PPARα, (D) SREBP‐1c, (E) FASN, (F) TNF‐α, (G) IL‐6, (H) NF‐κB p65, (I) SOD1, (J) GPX1, (K) Col1a1, and (L) TGF‐β1.

As shown in Figure 3C–3E, the mRNA expression levels of *de novo* lipogenesis‐related genes *SREBP‐1c* and *FASN* were significantly elevated in the T2DM group compared to the NC group (*p* < 0.05). In contrast, the expression of *PPARα*, a key gene responsible for fatty acid β‐oxidation, was substantially downregulated (*p* < 0.05). This pattern indicates a severe disruption of lipid metabolic homeostasis in the liver of T2DM mice. Post‐DPS treatment, the hepatic expression of both *SREBP‐1c* and *FASN* was significantly lower in the DPSL and DPSH groups than in the T2DM group, showing a dose‐dependent reduction (*p* < 0.05). Concurrently, the suppression of *PPARα* expression was notably reversed by DPS, with levels in the high‐dose group largely returning to normal. These results suggest that DPS effectively improves hepatic lipid metabolic dysfunction in diabetic mice by inhibiting lipid synthesis and simultaneously enhancing fatty acid oxidation.

Figure 3 F–3H reveal that, relative to the NC group, mRNA levels of the proinflammatory cytokines *TNF‐α* and *IL‐6*, as well as the key inflammatory transcription factor *NF‐κB p65*, were all significantly increased in the liver of the T2DM group (*p* < 0.05), indicating a profound hepatic inflammatory response induced by diabetes. DPS intervention significantly suppressed the transcriptional levels of these inflammatory mediators, with a more pronounced inhibitory effect observed in the high‐dose DPSH group compared to the low‐dose group, demonstrating a clear dose dependency (*p* < 0.05). These observations imply that DPS effectively alleviates diabetes‐associated hepatic inflammatory injury by downregulating the expression of inflammatory genes.

As presented in Figure 3I,J, the mRNA expression of the critical antioxidant enzymes *SOD1* and *GPX1* was significantly downregulated in the T2DM group compared to the NC group (*p* < 0.05), suggesting a transcriptional suppression of the hepatic antioxidant defense system under diabetic conditions. This alteration was significantly reversed by DPS treatment, which upregulated the expression of both *SOD1* and *GPX1*. Notably, the restorative effect was substantially stronger in the high‐dose DPSH group, exhibiting a distinct dose‐dependent pattern (*p* < 0.05). These results indicate that DPS can restore hepatic redox homeostasis and mitigate oxidative stress damage by enhancing the gene transcription of these antioxidant enzymes.

Figure 3 K and 3 L illustrate that the mRNA expression levels of *Col1a1*, a key extracellular matrix component, and the pro‐fibrotic core factor *TGF‐β1* were significantly elevated in the T2DM group versus the NC group (*p* < 0.05). This points toward an activation of pro‐fibrotic signaling pathways and an increased risk of matrix deposition in the diabetic liver. DPS administration significantly reversed this pathological shift, downregulating the expression of both *Col1a1* and *TGF‐β1* (*p* < 0.05). These data demonstrate that DPS can effectively delay or attenuate the progression of liver fibrosis in T2DM mice by inhibiting the transcription of pro‐fibrotic genes.

Further validation via Western blot analysis confirmed significant alterations in the expression of the AhR protein and the activation status of the NF‐κB signaling pathway in the livers of HFD/STZ‐induced T2DM mice. DPS intervention effectively counteracted these abnormalities. Specifically, hepatic AhR protein expression was markedly reduced in the T2DM group compared to the NC group (*p* < 0.05), indicating a suppression of the AhR signaling pathway in the diabetic state. After DPS treatment, hepatic AhR protein abundance was significantly restored in both the DPSL and DPSH groups relative to the T2DM group (*p* < 0.05). The AhR level in the high‐dose DPSH group recovered to near‐normal levels, suggesting that DPS can restore AhR expression in a dose‐dependent manner (Figure 4A). Conversely, the phosphorylation ratio of NF‐κB p65 (p‐p65/p65) was significantly increased in the T2DM group compared to the NC group (*p* < 0.05), indicating an overactivation of the NF‐κB‐mediated inflammatory pathway concomitant with hepatic steatosis and inflammatory injury in HFD/STZ‐induced T2DM mice. DPS treatment significantly lowered the p‐p65/p65 ratio (*p* < 0.05), with the high‐dose group showing a more potent inhibitory effect, essentially normalizing this parameter (Figure 4B). In summary, DPS treatment was associated with increased hepatic AhR expression and reduced NF‐κB p65 phosphorylation in diabetic mice. These observations indicate coordinated changes in AhR‐ and NF‐κB‐related markers, rather than direct evidence that AhR activation mediated the anti‐inflammatory effects of DPS.

**FIGURE 4:**
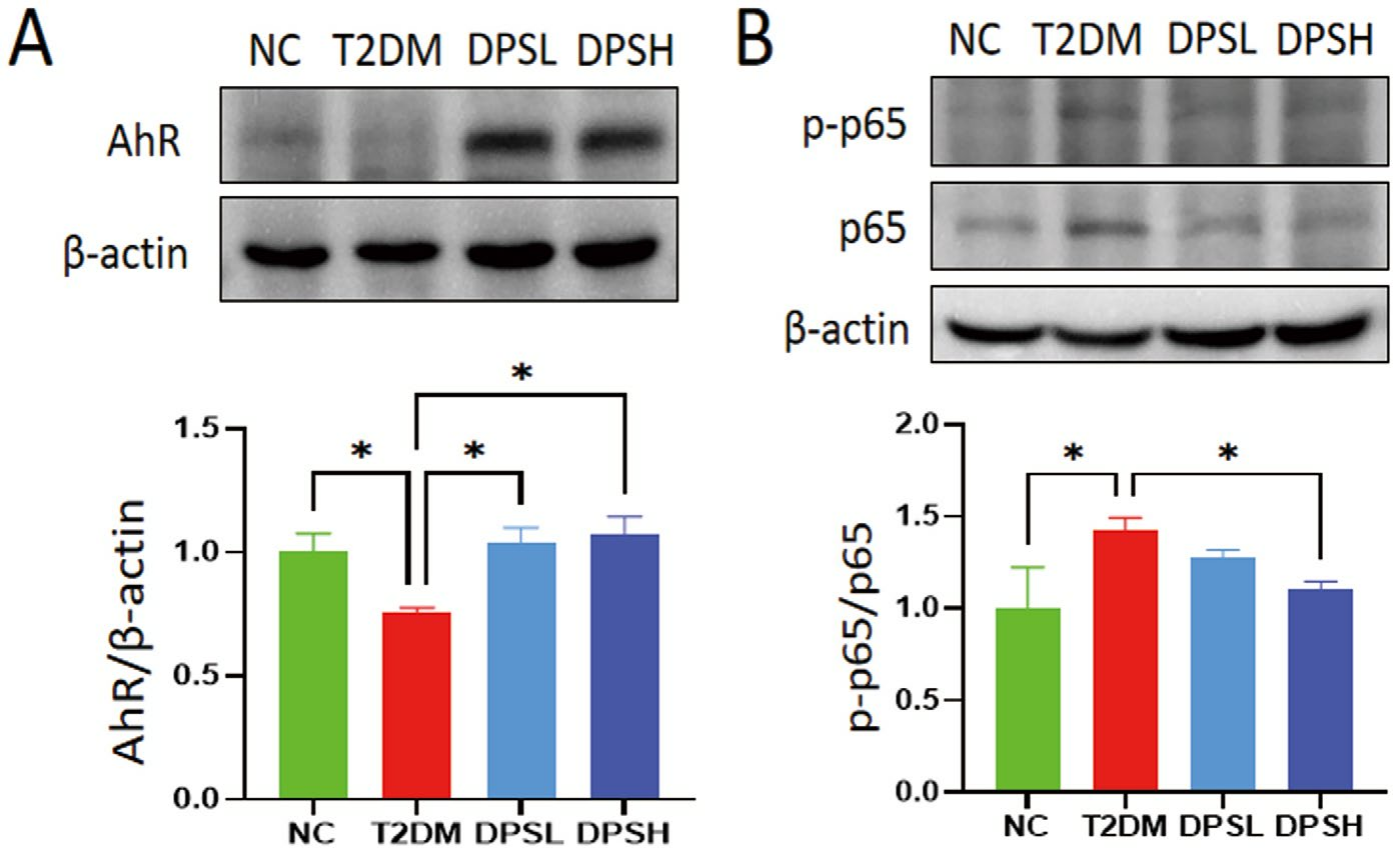
Western blot analysis of the AhR/NF‐κB axis (*n* = 3). Hepatic protein extracts were probed for: (A) AhR and (B) phosphorylated versus total NF‐κB p65. β‐Actin was used to verify equal loading. Bar charts represent the relative optical density of the bands. *p < 0.05 for the comparisons indicated by brackets. *p < 0.05 for the comparisons indicated by brackets.

These findings show that DPS treatment was associated with lower hepatic TDO2 expression and higher AhR expression in diabetic mice. These coordinated changes are consistent with altered TDO2/AhR‐related signaling but do not establish a causal regulatory relationship.

### 3.4 DPS Reverses Tryptophan‐Kynurenine Metabolic Disorder in T2DM Mice

UPLC‐MS/MS analysis revealed that a high‐fat diet combined with STZ successfully disrupted the tryptophan‐kynurenine metabolic pathway in mice, whereas allulose intervention significantly ameliorated this abnormality. Compared with the NC group, serum Trp levels in the T2DM group were significantly decreased (*p* < 0.05), indicating disordered Trp metabolism under diabetic conditions. After DPS intervention, both the DPSL and DPSH groups showed markedly elevated Trp levels compared with the T2DM group (*p* < 0.05). The high‐dose group (DPSH) exhibited Trp levels close to those of the NC group, with no significant difference compared with the low‐dose group (*p* > 0.05) (Figure 5A). Serum Kyn levels in the T2DM group were significantly higher than those in the NC group (*p* < 0.05). DPS treatment significantly reduced Kyn levels in diabetic mice; both the DPSL and DPSH groups showed a pronounced decrease compared with the T2DM group (*p* < 0.05). The reduction was more prominent in the high‐dose group, with a statistically significant difference compared with the low‐dose group (*p* < 0.05) (Figure 5B). Further analysis of the ratio of Kyn to Trp (Kyn/Trp, Figure 5C) revealed that the Kyn/Trp ratio was significantly increased in the T2DM group (*p* < 0.05), suggesting enhanced activity of the IDO/TDO pathway (Figure 5C). DPS intervention significantly suppressed this increase; both the DPSL and DPSH groups exhibited markedly lower ratios than the T2DM group (*p* < 0.05), with the high‐dose group showing a further reduction, approaching normal control levels. Serum IPA levels in the T2DM group were significantly lower than those in the NC group (*p* < 0.05). DPS treatment notably increased IPA levels; both the DPSL and DPSH groups showed significant elevation compared with the T2DM group (*p* < 0.05). Although the high‐dose group exhibited higher IPA levels than the low‐dose group, the difference did not reach statistical significance (*p* > 0.05) (Figure 5D).

**FIGURE 5:**
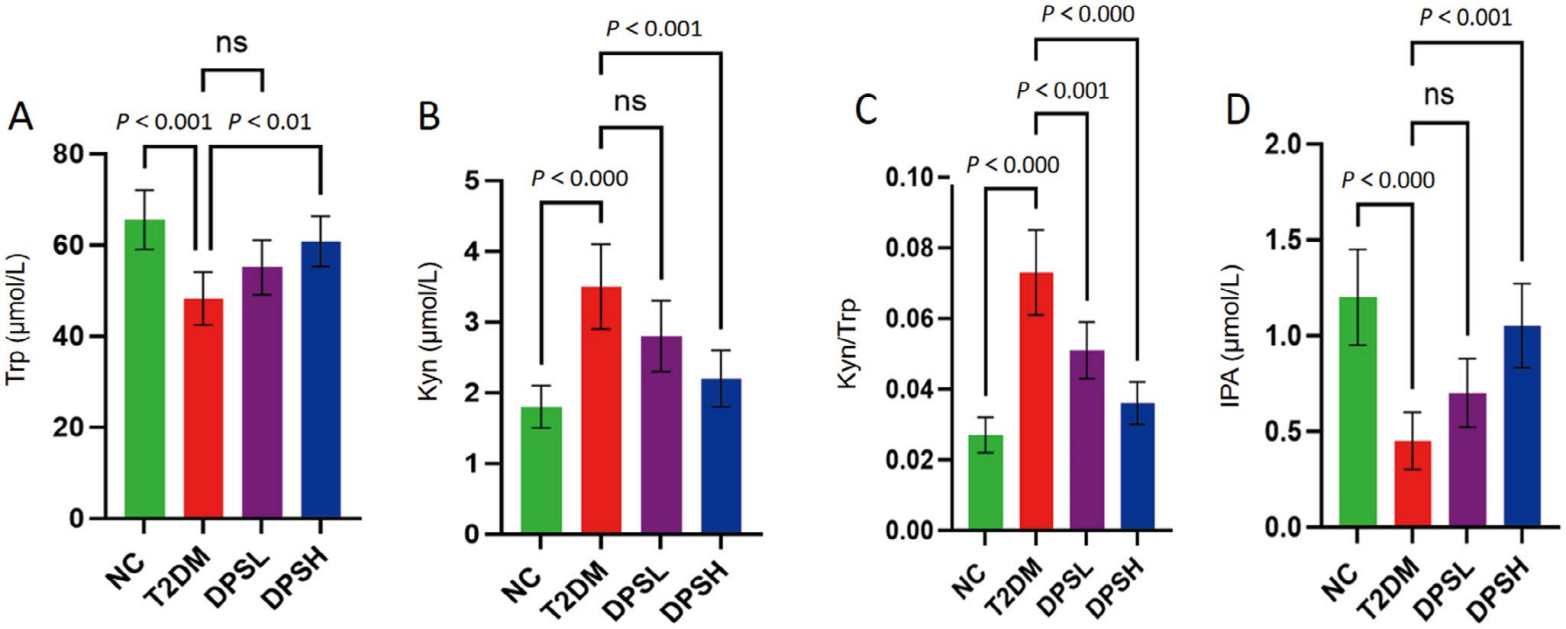
Effects of DPS on serum tryptophan and its metabolites in T2DM mice (*n* = 6). Serum levels of Trp (A), Kyn (B), the Kyn/Trp ratio (C), and IPA (D) in mice were analyzed using UPLC‐MS/MS.

High‐dose DPS improved the measured circulating tryptophan‐related metabolic abnormalities. Whether these changes contributed directly to the observed hepatic benefits requires further investigation.

### 3.5 DPS Attenuates Intestinal Barrier Dysfunction and Re‐Establishes Tight Junction Integrity in T2DM Mice

To evaluate the protective effect of DPS, we first examined the histological architecture of the intestinal mucosa via H&E staining (Figure 6A). In the NC group, the intestinal lining maintained structural integrity, characterized by elongated, well‐ordered villi and distinct crypts. Conversely, the T2DM group exhibited severe mucosal deterioration, manifested by stunted, disorganized villi and localized epithelial desquamation. These pathological changes signify a compromised intestinal physical barrier. Notably, DPS administration effectively reversed these structural abnormalities. Both the DPSL and DPSH groups displayed a recovery in villus height and alignment, with the high‐dose group (DPSH) demonstrating a restorative effect that nearly transitioned back to a healthy phenotype.

**FIGURE 6:**
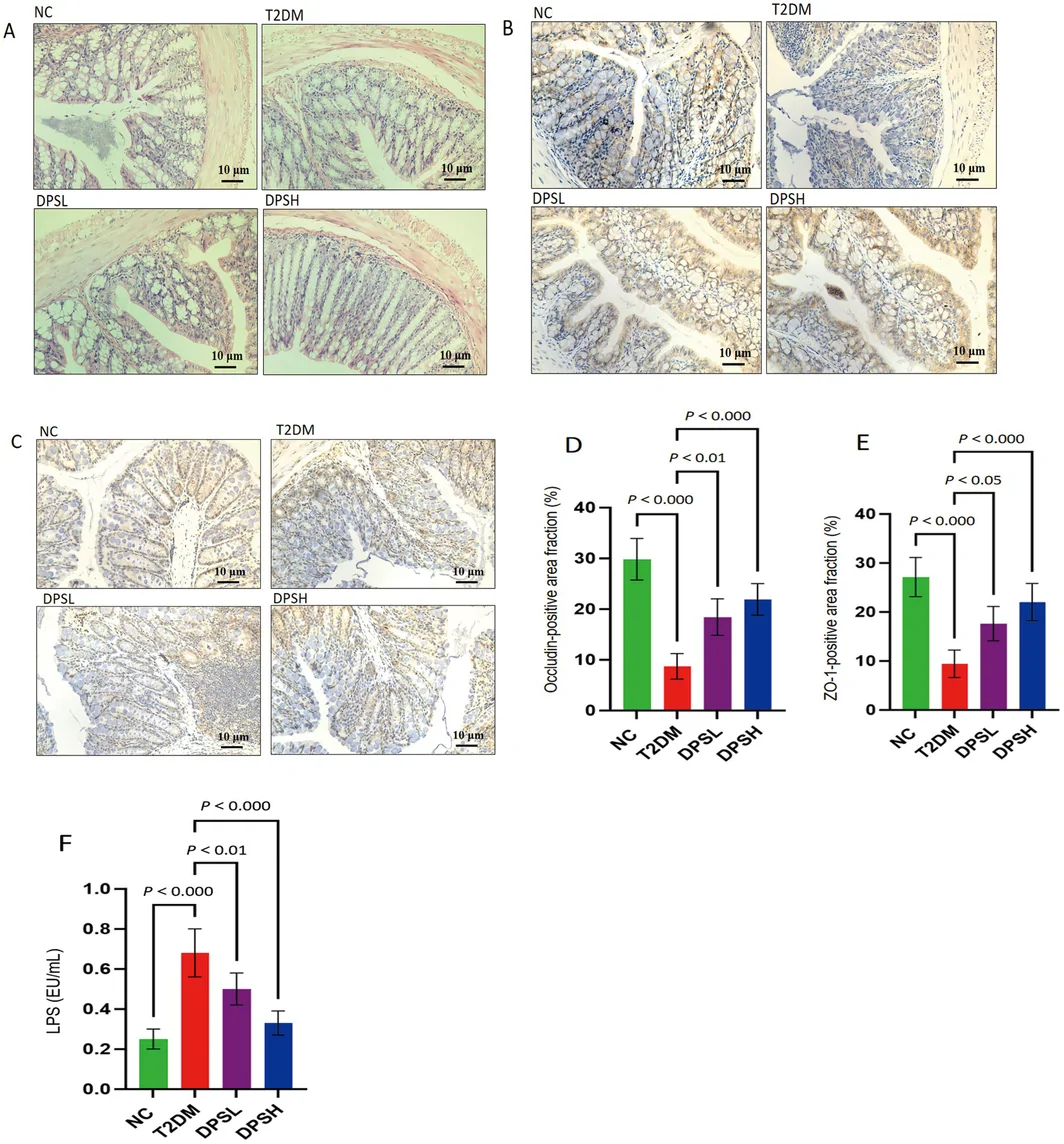
DPS treatment is associated with changes in intestinal morphology, tight‐junction protein immunoreactivity, and serum LPS levels in HFD/STZ‐induced T2DM mice (*n* = 3). (A) Representative hematoxylin and eosin (H&E)‐stained intestinal sections from the NC, T2DM, low‐dose DPS (DPSL), and high‐dose DPS (DPSH) groups. (B, C) Representative immunohistochemical staining for Occludin (B) and ZO‐1 (C) in intestinal epithelial tissue; brown staining indicates positive immunoreactivity. (D, E) Quantification of the Occludin‐positive area fraction (D) and ZO‐1‐positive area fraction (E). (F) LPS concentrations.

The functional integrity of the intestinal barrier is highly dependent on the expression of tight junction (TJ) proteins. As shown by immunohistochemical analysis (Figure 6B,C), the NC group exhibited robust and continuous brown‐positive staining for ZO‐1 and Occludin along the apical borders of the intestinal epithelium. In the T2DM group, these signals were markedly attenuated and fragmented, reflecting a loss of junctional complexity. However, DPS treatment significantly bolstered the expression of these proteins. In the DPSH group, the intensity and continuity of ZO‐1 and Occludin were substantially recovered, suggesting that DPS reinforces the molecular “seal” between intestinal epithelial cells.

To confirm whether the restoration of the physical barrier translated into improved barrier function, we quantified serum LPS concentrations (Figure 6D). Consistent with the structural damage observed, serum LPS levels in T2DM mice were drastically elevated compared to the NC group (*p* < 0.05), indicative of increased intestinal permeability and subsequent endotoxin leakage into the circulation. DPS intervention successfully suppressed this elevation in a dose‐dependent manner. Specifically, the DPSH group exhibited a significant decline in LPS levels (*p* < 0.05 vs. T2DM), highlighting the efficacy of DPS in mitigating “leaky gut” symptoms and reducing systemic inflammation associated with T2DM.

Together, the intestinal histological, tight‐junction immunostaining, and serum LPS findings were consistent with treatment‐associated differences in intestinal barrier‐related indices. They do not establish that improved barrier function mediated the hepatic or systemic effects observed after DPS administration.

### 3.6 DPS‐Associated Alterations in Gut Microbiota Composition in T2DM Mice

16S rRNA gene sequencing was performed in the NC, T2DM, and high‐dose DPS groups to describe group‐associated differences in gut microbial composition. Alpha‐diversity indices differed among the groups (Figure 7A). Ordination analysis also showed separation in overall community profiles between the NC and T2DM groups, with the DPS group occupying a distinct position in the ordination space (Figure 7B). These findings indicate differences in microbial community composition among groups; they should not be interpreted as evidence that DPS restored a healthy microbiota or corrected gut ecological homeostasis.

**FIGURE 7:**
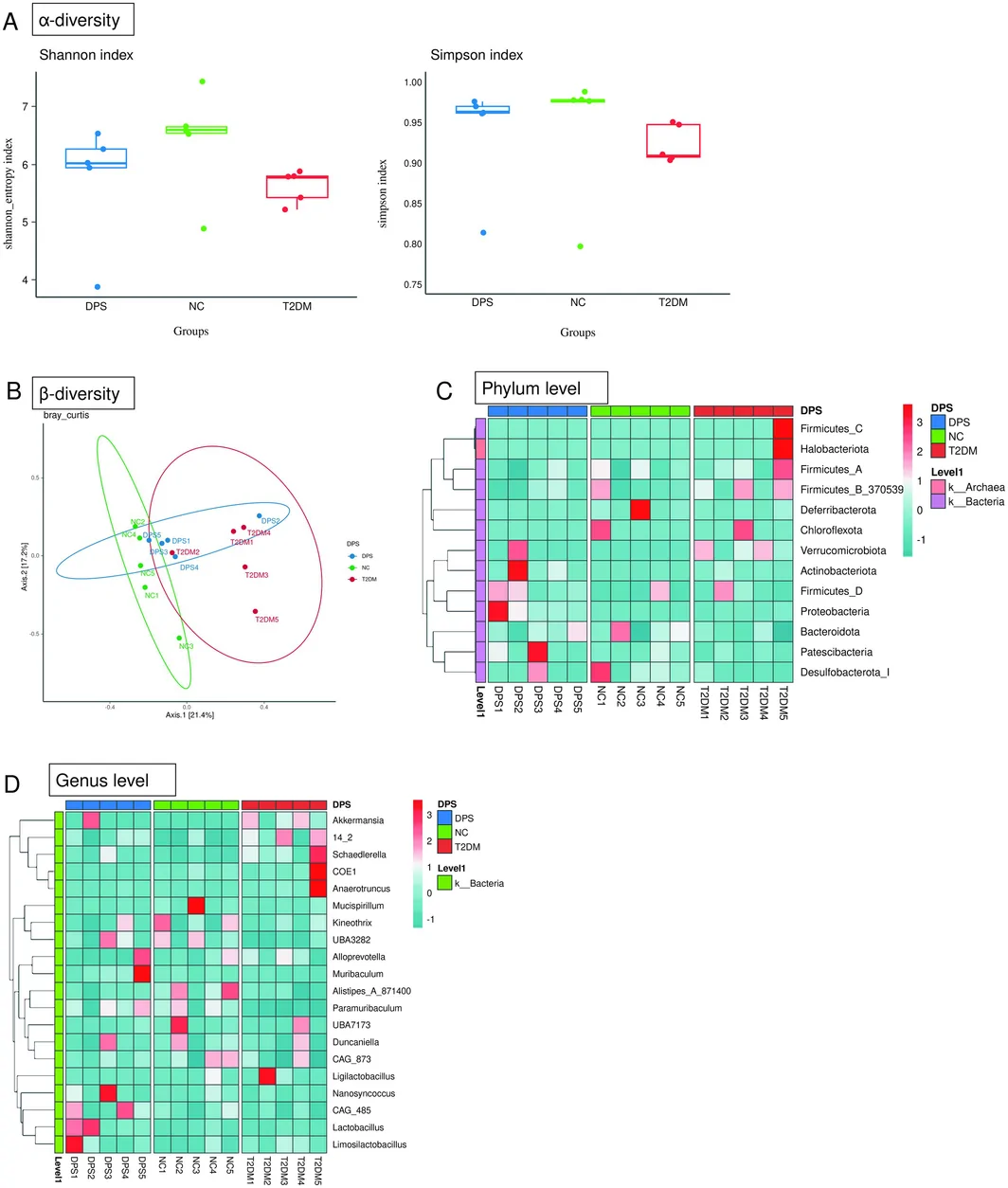
DPS‐associated alterations in gut microbial community profiles in T2DM mice (*n* = 5). (A) Microbial α‐diversity indices, assessing the internal variety and distribution within each group. (B) β‐diversity assessment using PCoA, reflecting the global shifts in microbiota architecture between NC, T2DM, and DPS‐treated mice. (C) Phylum‐level heatmap and (D) Genus‐level heatmap demonstrating the compositional landscape and abundance fluctuations of core bacterial taxa. The color gradient represents the log‐transformed or relative abundance levels, with clustering analysis identifying shared microbial trends.

The taxonomic landscape at the phylum level was dominated by Firmicutes, Bacteroidota, Proteobacteria, and Actinobacteriota (Figure 7C). The T2DM group was characterized by a skewed Firmicutes/Bacteroidota ratio and an expansion of proinflammatory taxa, specifically Proteobacteria. Following DPS intervention, this taxonomic imbalance was mitigated; the relative proportions of Firmicutes and Bacteroidota were recalibrated, and the overrepresentation of Proteobacteria was significantly suppressed, steering the community profile back toward a healthy state.

At the genus level, 16S rRNA gene sequencing revealed group differences in the relative abundances of several bacterial genera (Figure 7D). Compared with the NC group, the T2DM group showed lower relative abundances of *Akkermansia*, *Lactobacillus*, *Ligilactobacillus*, *Limosilactobacillus*, *Alloprevotella*, and *Muribaculum*, together with higher relative abundances of *Alistipes*, *Parabacteroides*, *Anaerotruncus*, *Mucispirillum*, and *Sutterella*. In the DPS‐treated group, the relative abundances of several of these genera shifted toward those observed in the NC group. These data demonstrate DPS‐associated differences in gut microbial composition; however, genus‐level 16S rRNA gene sequencing does not establish microbial metabolic capacity or demonstrate that any specific genus produced IPA, regulated tryptophan metabolism, or mediated the observed hepatic effects.

### 3.7 DPS‐Associated Changes in Serum Metabolomic Profiles in T2DM Mice

Untargeted serum metabolomics was performed in the NC, T2DM, and high‐dose DPS (DPSH; hereafter referred to as DPS) groups to characterize treatment‐associated differences in serum metabolic profiles. Unsupervised PCA showed separation among the three groups in both positive‐ and negative‐ion modes (Figure 8A), indicating differences in overall metabolite patterns. Pairwise OPLS‐DA models were subsequently established for the NC versus T2DM and DPS versus T2DM comparisons (Figure 8B). All models were evaluated by seven‐fold cross‐validation and 200‐permutation testing.

**FIGURE 8:**
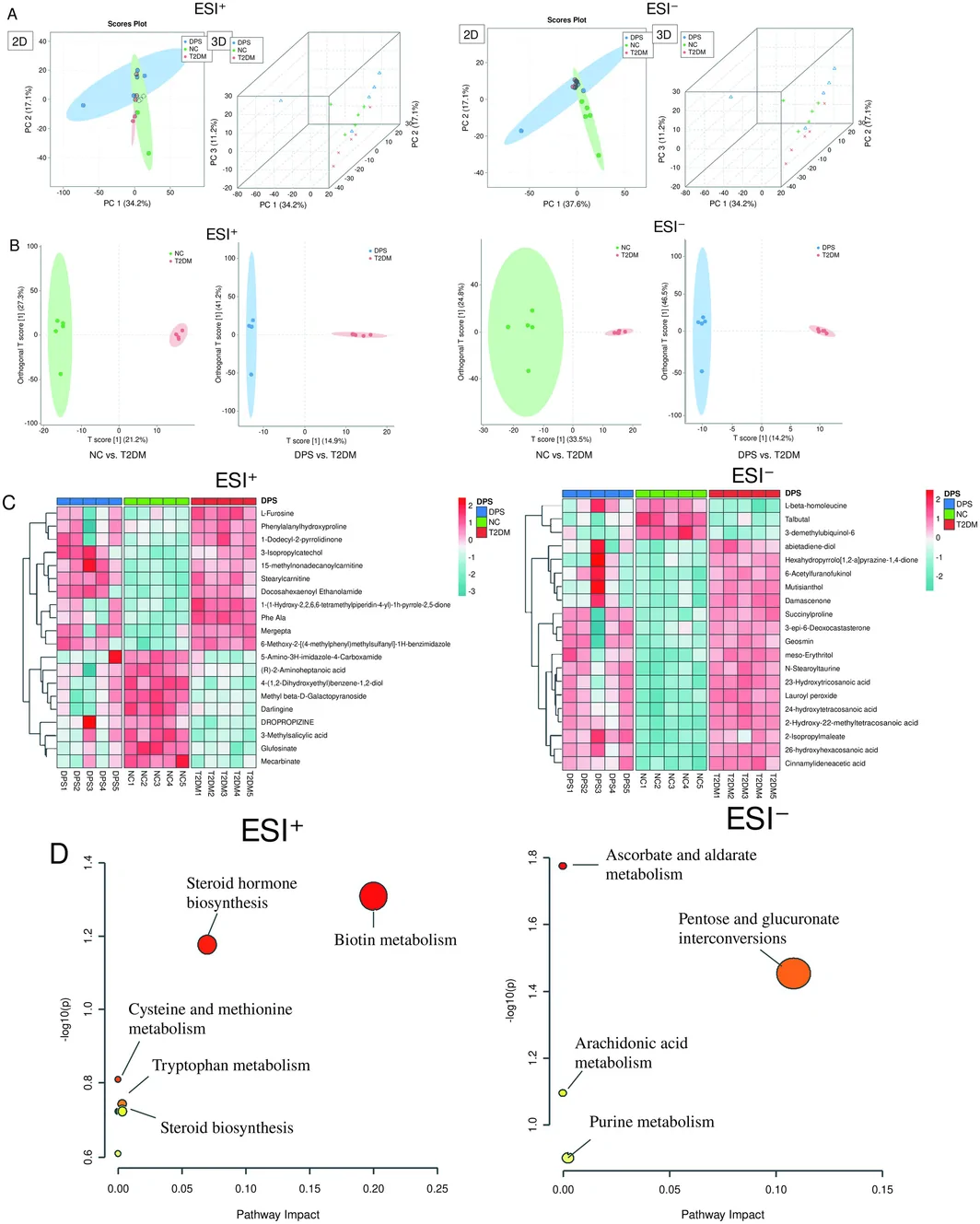
Integration of serum metabolic profiling and KEGG enrichment analysis (*n* = 5). (A) PCA score plots visualizing the global shift in metabolic phenotypes across NC, T2DM, and DPS‐treated mice. (B) Discriminant analysis (OPLS‐DA) providing clear binary comparisons to reveal the regulatory capacity of DPS on the serum metabolome. (C) Dendrogram‐linked heatmaps showing the divergent profiles of screened metabolites based on relative intensity. (D) KEGG annotation and enrichment analysis of differential serum metabolites. Enriched pathway terms represent database‐based annotations and do not directly demonstrate pathway activity, with bubble morphology and color reflecting impact and *p*‐values, respectively.

In positive‐ion mode, the NC versus T2DM model showed R^2^X(cum) = 0.674, R^2^Y(cum) = 0.998, and Q^2^(cum) = 0.895, with permutation‐test pR^2^Y = 0.015 and pQ^2^ = 0.010. The DPS versus T2DM model showed R^2^X(cum) = 0.725, R^2^Y(cum) = 0.990, and Q^2^(cum) = 0.666, with pR^2^Y = 0.035 and pQ^2^ = 0.030. In negative‐ion mode, the NC versus T2DM model yielded R^2^X(cum) = 0.679, R^2^Y(cum) = 0.999, and Q^2^(cum) = 0.914, with pR^2^Y = 0.020 and pQ^2^ = 0.010, whereas the DPS versus T2DM model yielded R^2^X(cum) = 0.727, R^2^Y(cum) = 0.999, and Q^2^(cum) = 0.740, with pR^2^Y = 0.010 and pQ^2^ = 0.020. These internally validated models support differences in serum metabolic profiles between the compared groups. Notably, the positive‐ion DPS versus T2DM model had a lower Q^2^ value than the NC versus T2DM model; therefore, this comparison was interpreted cautiously.

KEGG annotation of the differential serum metabolites identified enrichment of terms related to biotin metabolism, steroid hormone biosynthesis, cysteine and methionine metabolism, ascorbate and aldarate metabolism, arachidonic acid metabolism, and purine metabolism (Figure 8 CD). These results indicate that the metabolites differing between groups were annotated to several metabolism‐related pathways. However, untargeted metabolomics and database‐based pathway enrichment do not directly measure pathway flux, enzyme activity, or causal pathway regulation. Therefore, these pathway annotations should be interpreted as candidate biological contexts for the observed metabolite differences and require targeted metabolite quantification and functional validation in future studies.

In summary, serum metabolomics identified marked differences in metabolic features between T2DM mice and DPS‐treated mice. When considered together with the 16S rRNA gene sequencing results, these data demonstrate concurrent treatment‐associated changes in serum metabolite profiles and gut microbial composition. They do not establish functional links between individual taxa and specific metabolites, nor do they demonstrate that the observed microbial or metabolic changes caused the hepatic benefits of DPS.

## 4 Discussion

This study evaluated treatment‐associated changes following DPS administration in HFD/STZ‐induced T2DM mice. DPS‐treated mice showed differences in liver histology, serum lipid and aminotransferase profiles, inflammatory indices, intestinal barrier‐related markers, and selected hepatic molecular markers compared with untreated diabetic mice. Fasting blood glucose was used to confirm successful diabetes induction; however, longitudinal glycemic outcomes, glucose tolerance, insulin sensitivity, and glycated hemoglobin were not assessed. Therefore, the present study does not provide direct evidence that DPS improved glycemic control in this model.

Our study demonstrates that DPS exhibits significant hepatoprotective, lipid‐lowering, and anti‐inflammatory effects, which aligns with previous findings on rare sugars improving metabolic disorders and insulin resistance (Zhang et al. 2023). Pathological and biochemical analyses confirm that DPS effectively reversed hepatic steatosis and inflammatory cell infiltration in T2DM mice while reducing serum transaminase and lipid levels. At the molecular mechanism level, earlier studies in human and animal models of obesity have indicated that DPS reduces body fat accumulation by inhibiting the activity of hepatic lipogenic enzymes (such as FASN and ACC; Zhou et al. 2026). In the present study, we also observed that DPS significantly downregulated the expression of key genes involved in hepatic lipid synthesis (SREBP‐1c, FASN) and upregulated genes associated with fatty acid oxidation (PPARα; Lee et al. 2025; Shin et al. 2026). This suggests that DPS does not merely inhibit lipid synthesis but corrects lipid metabolism imbalance from the source through a dual mechanism of “increasing expenditure and reducing intake”, thereby achieving a fundamental therapeutic effect.

Inhibition of inflammatory responses constitutes another key mechanism through which DPS exerts its protective effects. While recent studies have confirmed that DPS alleviates systemic “metainflammation” by modulating adipose tissue macrophages (Figueiredo et al. 2022; Jia et al. 2025), this research focuses specifically on the molecular signal transduction within the liver, providing deeper mechanistic insights. Our results show that DPS significantly suppresses the phosphorylation‐driven activation of the hepatic NF‐κB signaling pathway, thereby reducing the release of downstream pro‐inflammatory cytokines (TNF‐α, IL‐6, IL‐1β; Aili et al. 2025; Preechasuk et al. 2023). This experimental evidence not only corroborates the anti‐inflammatory properties of DPS but also aligns closely with the findings from our network pharmacology analysis, which predicted that “DPS modulates inflammatory responses through the synergistic targeting of multiple nodes, including NF‐κB p65 (Wang et al. 2024)”. The network pharmacology methods and exploratory findings are provided in Methods S4 and Figure S2. These computational predictions offer hypotheses for further investigation but do not establish direct molecular targets or a causal mechanism of DPS action (Xiao et al. 2025).

This study identified coordinated changes in intestinal barrier‐related indices, gut microbial composition, circulating tryptophan metabolites, and hepatic inflammatory signaling after DPS treatment. Previous studies have reported that DPS may alter gut microbial composition in high‐fat diet‐induced metabolic disorders (Escalante‐Araiza et al. 2022; Lee et al. 2024). In the present study, DPS treatment was accompanied by relative increases in *Akkermansia* and *Lactobacillus*, improved intestinal morphology, increased ZO‐1 and Occludin expression, and lower serum LPS concentrations. These findings are consistent with the reported associations of *Akkermansia* and intestinal barrier‐related indices with metabolic health (Cani et al. 2022; Ye et al. 2026), and with the proposed prebiotic‐like properties of D‐allulose (Adolphus et al. 2025). Gut microbiota are important participants in host tryptophan metabolism (Su et al. 2022). *Lactobacillus* species can generate indole derivatives with AhR‐ligand activity (Zelante et al. 2013), and Akkermansia has been linked to tryptophan catabolism in experimental systems (Yin et al. 2021). In addition, indole derivatives, including IPA and ILA, have been associated with anti‐inflammatory effects in previous studies (Madella et al. 2022; Roager and Licht 2018; Salminen 2023). In our dataset, the microbial compositional changes observed after DPS treatment co‐occurred with higher serum IPA levels, lower Kyn levels, and a lower Kyn/Trp ratio (Wen et al. 2025). However, because 16S rRNA gene sequencing provides taxonomic rather than direct functional evidence, these findings do not establish that the observed microbial taxa produced the altered circulating metabolites or that DPS‐induced microbial changes directly redirected tryptophan metabolism (Pabst et al. 2023).

TDO2 is a rate‐limiting enzyme in hepatic tryptophan‐kynurenine metabolism, and its expression may be influenced by inflammatory stimuli (Fujiwara et al. 2022). Disturbances in tryptophan‐kynurenine metabolism have also been linked to insulin resistance and inflammatory states (Oxenkrug 2013). In the present study, DPS treatment was associated with lower hepatic TDO2 expression, higher hepatic AhR expression, reduced NF‐κB p65 phosphorylation, and improvement in serum tryptophan‐related metabolic indices. These observations provide a biologically plausible framework for further investigation of interactions among inflammation, TDO2‐related metabolism, indole metabolites, and AhR‐related signaling (Cani et al. 2022; Laurans et al. 2018; Zhang et al. 2022). Nevertheless, the current study did not determine whether improved intestinal barrier‐related indices or microbial compositional changes preceded the changes in TDO2, circulating IPA, AhR expression, or hepatic inflammation. Therefore, these results should be interpreted as coordinated treatment‐associated findings rather than evidence for a causal gut microbiota–tryptophan–AhR pathway.

The integrated 16S rRNA gene sequencing and untargeted serum metabolomics analyses showed concurrent alterations in gut microbial composition and systemic metabolic profiles after DPS treatment. The shift in serum metabolite patterns toward those of the NC group coincided with relative changes in several bacterial genera, including *Akkermansia*, *Lactobacillus*, *Ligilactobacillus*, *Alistipes*, and *Parabacteroides* (Fan and Pedersen 2021; Llauradó et al. 2025). Previous studies have associated *Akkermansia* with metabolic health and intestinal barrier function (Depommier et al. 2019) and have identified lactic acid bacteria as potential contributors to tryptophan‐derived indole metabolism (Roager and Licht 2018). However, the present study did not directly link individual taxa to specific serum metabolites or demonstrate microbial production of IPA. Thus, the concordance between the microbiota and metabolomics findings should not be interpreted as proof that the observed microbial changes caused the altered host metabolic profile.

KEGG enrichment analysis identified pathways related to tryptophan metabolism, arachidonic acid metabolism, ascorbate and aldarate metabolism, biotin metabolism, and steroid hormone biosynthesis. These pathways have been reported to interact with microbial composition and host inflammatory or metabolic status (Fu et al. 2025; Postler and Ghosh 2017; Zhao et al. 2025). Some serum metabolites altered in this study may also be influenced by microbial or host–microbial co‐metabolism (Miyamoto et al. 2015; Yoshii et al. 2019). Nevertheless, the present taxonomic and metabolomic analyses cannot establish the directionality of these relationships or attribute metabolic changes to specific microbial genera. Collectively, the results support the hypothesis that DPS‐associated changes in gut microbial composition and systemic metabolism may be relevant to its beneficial effects in diabetic mice, but dedicated functional studies are needed to determine whether these changes mediate hepatic protection.

This study has several limitations. First, the HFD/STZ model reproduces selected metabolic and hepatic abnormalities relevant to T2DM, but it does not fully reflect the biological heterogeneity, prolonged natural history, or full pathological spectrum of human T2DM‐associated MASLD. In particular, the combination of high‐fat feeding and chemically induced β‐cell injury may not mirror the relative contributions of diet, genetics, lifestyle, and progressive liver injury in patients. The modest sample size (*n* = 6 per group) also limits statistical precision and the generalizability of the findings. Therefore, the present results should be interpreted as preclinical observations from a single animal model. Future studies should assess DPS in complementary diet‐induced and/or genetic models, with longer intervention periods and evaluation of more advanced liver pathology, and should ultimately examine its relevance in human studies.

Second, although DPS treatment was associated with coordinated changes in gut microbial composition, circulating tryptophan metabolites, intestinal barrier‐related indices, and hepatic AhR/NF‐κB‐related markers, no causal relationship was established. Fecal microbiota transplantation, microbiota depletion, germ‐free animal experiments, and direct microbial functional analyses were not performed. Third, AhR expression was measured at the mRNA and protein levels, but AhR activity was not directly evaluated using pharmacological or genetic perturbation, nuclear‐translocation assays, or downstream functional readouts. In addition, the source of the altered circulating IPA and its direct role in hepatic signaling were not determined. Thus, the proposed gut microbiota–tryptophan–AhR relationship should be considered a hypothesis for future testing rather than a demonstrated mechanism.

## 5 Conclusion

In HFD/STZ‐induced T2DM mice with hepatic steatosis and inflammatory injury, DPS treatment was associated with lower serum aminotransferase activities, less hepatic steatosis and inflammatory histological changes, and more favorable lipid and inflammatory profiles. These changes occurred together with alterations in intestinal barrier‐related indices, gut microbial composition, circulating tryptophan metabolites, hepatic TDO2 and AhR expression, and NF‐κB p65 phosphorylation. The findings do not establish a causal gut microbiota–tryptophan–AhR mechanism. Because the HFD/STZ model does not fully reproduce the heterogeneity and natural history of human T2DM‐associated MASLD, the results should not be directly extrapolated to patients.

## Author Contributions

**Liang Wu:** conceptualization, investigation, funding acquisition, writing – original draft, writing – review and editing, methodology, visualization, software, data curation, supervision, resources. **Xueyun Dong:** conceptualization, investigation, methodology, validation, writing – original draft, software, data curation. **Wen Sun:** conceptualization, investigation, writing – original draft, funding acquisition, software, formal analysis, project administration, writing – review and editing. **Hao Xu:** conceptualization, investigation, methodology, validation, visualization, resources, data curation, project administration. **Yunhan Xie:** conceptualization, investigation, visualization, validation, methodology. **Xuehui Liu:** conceptualization, investigation, validation, methodology, software. **Xinyu Liu:** conceptualization, investigation, data curation, resources. **Asmaa Ali:** conceptualization, investigation, writing – original draft, writing – review and editing. **Keke Shao:** conceptualization, investigation, writing – review and editing, project administration, formal analysis, software, resources, supervision.

## Funding

This work was supported by the Scientific Research Project of Yancheng Municipal Health Commission (Grant No. YK2024116), the Open Foundation of the Jiangsu Provincial Key Laboratory of Laboratory Medicine (Grant No. JSKLM‐Y‐2024‐019), and the Zhenjiang Social Development Project (Grant No. SH2025055).

## Disclosure

*Author approval and responsibility statement*: All authors have read and approved the final version of the manuscript. Liang Wu, as the corresponding author and manuscript guarantor, had full access to all of the data in this study and takes complete responsibility for the integrity of the data and the accuracy of the data analysis.

## Ethics Statement

All animal procedures were reviewed and approved by the Animal Ethics Committee of Jiangsu University (Approval No. UJS‐IACUCAP‐2024032261). All experiments were conducted in accordance with the National Institutes of Health Guide for the Care and Use of Laboratory Animals (NIH Publications No. 8023, revised 1978).

## Conflicts of Interest

The authors declare no conflicts of interest.

## Supporting information


**Figure S1:** Internal validation of pairwise OPLS‐DA models for serum metabolomic profiling. OPLS‐DA models were generated in positive‐ion (ESI+) and negative‐ion (ESI−) modes for comparisons between NC and T2DM mice and between the high‐dose DPS group (DPS) and T2DM mice. Each row shows the model overview (left), 200‐permutation test (middle), and model performance parameters [R^2^X(cum), R^2^Y(cum), and Q^2^(cum)] (right). (A) ESI+, NC versus T2DM: R^2^X(cum) = 0.674, R^2^Y(cum) = 0.998, Q^2^(cum) = 0.895; pR^2^Y = 0.015 and pQ^2^ = 0.010. (B) ESI+, DPS versus T2DM: R^2^X(cum) = 0.725, R^2^Y(cum) = 0.990, Q^2^(cum) = 0.666; pR^2^Y = 0.035 and pQ^2^ = 0.030. (C) ESI−, NC versus T2DM: R^2^X(cum) = 0.679, R^2^Y(cum) = 0.999, Q^2^(cum) = 0.914; pR^2^Y = 0.020 and pQ^2^ = 0.010. (D) ESI−, DPS versus T2DM: R^2^X(cum) = 0.727, R^2^Y(cum) = 0.999, Q^2^(cum) = 0.740; pR^2^Y = 0.010 and pQ^2^ = 0.020. All models were assessed using seven‐fold cross‐validation and 200‐permutation testing.
**Figure S2:** Computational decoding of the pharmacological mechanisms of DPS against diabetic liver injury via network analysis. (A) Venn diagrammatic representation identifying the molecular intersection between DPS‐responsive proteins and the pathological signatures of T2DM and NAFLD. (B) Functional interactome constructed via the STRING database, characterizing the PPI landscape among common targets. (C) Hub gene topography, prioritizing core regulatory nodes based on topological centrality; node dimensions and chromaticity denote the relative influence (degree) within the network. (D) GO‐based functional profiling, categorizing shared targets into Biological Process, Cellular Component, and Molecular Function domains, with significance levels determined by ‐log_10_ (*p*‐value). (E) KEGG pathway bubble plot, mapping the enriched signaling routes influenced by DPS. The sphere diameter correlates with the gene count, while the color spectrum highlights the statistical enrichment potency.
**Table S1:** Primer sequence.
**Table S2:** Antibodies used for western blot analysis.
**Table S3:** Internal validation statistics for OPLS‐DA models of serum metabolomics.

## Data Availability

The data that support the findings of this study are available from the corresponding author upon reasonable request.
