# Supplementary material for: D‐Psicose Attenuates Hepatic Steatosis and Inflammatory Injury in HFD/STZ‐Induced T2DM Mice and Is Associated With Alterations in Gut Microbiota, Tryptophan Metabolism, and Hepatic AhR‐Related Signaling
Source: Food Sci Nutr. 2026 Oct 8;14(10):e72455. doi: 10.1002/fsn3.72455 (PMC13650261; doi:10.1002/fsn3.72455)
Supplement: Supplementary file 1 [file FSN3-14-e72455-s001.docx]

**Supplementary Methods S1:**

Table S1 Primer sequence

| Gene | Forward Primer | Reverse Primer |
| --- | --- | --- |
| *TDO2* | GCTGGAGTTTGGGAAGCTGA | CATGGCCACAGACACCTTCA |
| *AhR* | CCAAGGACAAGCCCAAGAGG | CAGAAGCGGTCCTTGGTGAT |
| *PPARα* | AGAGCCCCATCTGTCCTCTC | ACTGGTAGTCTGCAAAACCAAA |
| *SREBP-1c* | CTGTCGGGGTAGCGTCCT | GCTGGTAGATGTCGTTCACA |
| *FASN* | GGAGGTGGTGATAGCCGGTAT | TGGGTAATCCATAGAGCCCAG |
| *TNF-α* | CAGGCGGTGCCTATGTCTC | CGATCACCCCGAAGTTCAGTAG |
| *IL-6* | CCAAGAGGTGAGTGCTTCCC | CTGTTGTTCAGACTCTCTCCCT |
| *NF-κB p65* | ATGGCAGACGATGATCCCTAC | CGGAATCGAAATCCCCTCTGTT |
| *SOD1* | CAGACCTGCCTTACGACTATGG | CTCGGTGGCGTTGAGATTGTT |
| *GPX1* | CAGTTCGGACATCAGGAGAATG | GAGGGACGCCACATTCTCG |
| *Col1a1* | GCTCCTCTTAGGGGCCACT | CCACGTCTCACCATTGGGG |
| *TGF-β1* | CTCCCGTGGCTTCTAGTGC | GCCTTAGTTTGGACAGGATCTG |
| *β-actin* | GGCTGTATTCCCCTCCATCG | CCAGTTGGTAACAATGCCATGT |

**Supplementary Methods S2: Western blot analysis**

Liver tissues from individual mice were lysed on ice in RIPA buffer supplemented with PMSF and phosphatase inhibitors (Beyotime, Shanghai, China). The lysates were centrifuged at 12,000 × g for 15 min at 4 °C, and the supernatants were collected. Total protein concentrations were determined using a bicinchoninic acid assay kit (Beyotime). Equal amounts of protein (30 μg per lane) were separated by 10% sodium dodecyl sulfate-polyacrylamide gel electrophoresis and transferred onto polyvinylidene difluoride membranes (0.45 μm; Millipore, USA).

Membranes were blocked with 1% bovine serum albumin in Tris-buffered saline containing 0.1% Tween-20 for 1 h at room temperature. Membranes were then incubated overnight at 4 °C with the primary antibodies listed in Table S2. After washing, the membranes were incubated with the appropriate horseradish peroxidase-conjugated secondary antibody for 1 h at room temperature. Protein bands were visualized using an enhanced chemiluminescence reagent (Vazyme, Nanjing, China).

Band intensities were quantified using ImageJ software after background subtraction. AhR protein abundance was normalized to β-actin. NF-κB p65 phosphorylation was expressed as the ratio of phospho-NF-κB p65 (Ser536) to total NF-κB p65. Each biological replicate represented liver tissue collected from an individual mouse.

**Table S2.** Antibodies used for western blot analysis.

| Target | Primary antibody | Catalog No. | Host species | Working dilution | Supplier |
| --- | --- | --- | --- | --- | --- |
| AhR | Anti-AhR | 67785-1-Ig | Mouse monoclonal | 1:1,000 | Proteintech Group, Wuhan, China |
| NF-κB p65 | Anti-NF-κB p65 | 80979-1-RR | Rabbit recombinant monoclonal | 1:5,000 | Proteintech Group, Wuhan, China |
| Phospho-NF-κB p65 (Ser536) | Anti-phospho-NF-κB p65 (Ser536) | 80379-2-RR | Rabbit recombinant monoclonal | 1:2,000 | Proteintech Group, Wuhan, China |
| β-actin | Anti-β-actin | 66009-1-Ig | Mouse monoclonal | 1:5,000 | Proteintech Group, Wuhan, China |
| Rabbit secondary antibody | HRP-conjugated goat anti-rabbit IgG (H+L) | SA00001-2 | Goat | 1:5,000 | Proteintech Group, Wuhan, China |
| Mouse secondary antibody | HRP-conjugated goat anti-mouse IgG (H+L) | SA00001-1 | Goat | 1:5,000 | Proteintech Group, Wuhan, China |

**Supplementary Methods S3: Untargeted Serum Metabolomics**

**Table S3.** Internal validation statistics for OPLS-DA models of serum metabolomics

| **Comparison** | **Ion mode** | **R²X(cum)** | **R²Y(cum)** | **Q²(cum)** | **Permutations** | **pR²Y** | **pQ²** |
| --- | --- | --- | --- | --- | --- | --- | --- |
| NC vs. T2DM | ESI+ | 0.674 | 0.998 | 0.895 | 200 | 0.015 | 0.010 |
| DPS vs. T2DM | ESI+ | 0.725 | 0.990 | 0.666 | 200 | 0.035 | 0.030 |
| NC vs. T2DM | ESI− | 0.679 | 0.999 | 0.914 | 200 | 0.020 | 0.010 |
| DPS vs. T2DM | ESI− | 0.727 | 0.999 | 0.740 | 200 | 0.010 | 0.020 |

**Figure S1.** Internal validation of pairwise OPLS-DA models for serum metabolomic profiling. OPLS-DA models were generated in positive-ion (ESI+) and negative-ion (ESI−) modes for comparisons between NC and T2DM mice and between the high-dose DPS group (DPS) and T2DM mice. Each row shows the model overview (left), 200-permutation test (middle), and model performance parameters [R²X(cum), R²Y(cum), and Q²(cum)] (right). (A) ESI+, NC versus T2DM: R²X(cum) = 0.674, R²Y(cum) = 0.998, Q²(cum) = 0.895; pR²Y = 0.015 and pQ² = 0.010. (B) ESI+, DPS versus T2DM: R²X(cum) = 0.725, R²Y(cum) = 0.990, Q²(cum) = 0.666; pR²Y = 0.035 and pQ² = 0.030. (C) ESI−, NC versus T2DM: R²X(cum) = 0.679, R²Y(cum) = 0.999, Q²(cum) = 0.914; pR²Y = 0.020 and pQ² = 0.010. (D) ESI−, DPS versus T2DM: R²X(cum) = 0.727, R²Y(cum) = 0.999, Q²(cum) = 0.740; pR²Y = 0.010 and pQ² = 0.020. All models were assessed using seven-fold cross-validation and 200-permutation testing.

**Supplementary Methods S4: Multi-target synergistic mechanisms of DPS against diabetic liver injury via network pharmacology**

We applied network pharmacology to map the therapeutic actions of DPS. The DPS structure (SMILES) was sourced from PubChem, and its potential targets were identified using PharmMapper, SwissTargetPrediction, and SEA. After normalizing targets to UniProt IDs, we identified overlapping genes between DPS targets and disease targets (T2DM/NAFLD) retrieved from GeneCards, OMIM, DisGeNET, and TTD. This intersection dataset was used to build a PPI network via STRING, which was then visualized in Cytoscape. Key hub genes were selected based on topological centrality (degree, betweenness, closeness). Furthermore, the functional roles of these targets were clarified through GO and KEGG pathway enrichment analyses, providing insight into the molecular mechanisms underlying the effects of DPS on diabetic liver injury.

To systematically elucidate the molecular mechanism by which DPS mitigates diabetic liver injury, we integrated the predicted pharmacological repertoire of DPS with clinical databases of T2DM, diabetic liver injury, and NAFLD. Bioinformatic intersection analysis identified 198 overlapping targets (Figure S1A), representing the core therapeutic interface of DPS. A PPI network was subsequently established using the STRING database to explore the functional crosstalk among these candidates (Figure S1B). Evaluation of topological centrality prioritized several high-degree hub genes, specifically STAT3, ESR1, RELA, HSP90AA1, TLR4, FN1, PTGS2, KDR, CYP2C9, and ALOX15, which function as critical regulatory nodes in inflammatory signaling, oxidative stress, and extracellular matrix remodeling (Figure S1C). Functional enrichment analysis further decoded the biological implications of this target pool; Gene Ontology (GO) mapping highlighted their involvement in modulating systemic immune responses, apoptotic cascades, and signal transduction at the cell membrane (Figure S1D). Concomitantly, KEGG pathway analysis revealed that these targets were significantly enriched in pivotal routes including insulin resistance, calcium signaling, and inflammatory pathways, alongside metabolic hubs such as arginine and proline metabolism (Figure S1E). Collectively, these findings suggest that DPS exerts its hepatoprotective effects through a synergistic multi-target strategy, reinforcing metabolic homeostasis and dampening inflammatory damage, which provides a robust theoretical foundation for its therapeutic application.

**Figure. S2.** Computational decoding of the pharmacological mechanisms of DPS against diabetic liver injury via network analysis. (A) Venn diagrammatic representation identifying the molecular intersection between DPS-responsive proteins and the pathological signatures of T2DM and NAFLD. (B) Functional interactome constructed via the STRING database, characterizing the PPI landscape among common targets. (C) Hub gene topography, prioritizing core regulatory nodes based on topological centrality; node dimensions and chromaticity denote the relative influence (degree) within the network. (D) GO-based functional profiling, categorizing shared targets into Biological Process, Cellular Component, and Molecular Function domains, with significance levels determined by -log_10_ (*P*-value). (E) KEGG pathway bubble plot, mapping the enriched signaling routes influenced by DPS. The sphere diameter correlates with the gene count, while the color spectrum highlights the statistical enrichment potency.
